# YWHAZ interacts with DAAM1 to promote cell migration in breast cancer

**DOI:** 10.1038/s41420-021-00609-7

**Published:** 2021-08-27

**Authors:** Jie Mei, Yan Liu, Xinqian Yu, Leiyu Hao, Tao Ma, Qiang Zhan, Yan Zhang, Yichao Zhu

**Affiliations:** 1 Department of Oncology, Wuxi Maternal and Child Health Hospital Affiliated to Nanjing Medical University, Wuxi, 214023 China; 2 Wuxi College of Clinical Medicine, Nanjing Medical University, Wuxi, 214023 China; 3 Department of Physiology, Nanjing Medical University, Nanjing, 211166 China; 4 Department of Breast Surgery, Wuxi Maternal and Child Health Hospital Affiliated to Nanjing Medical University, Wuxi, 214023 China; 5 Department of Gastroenterology, Wuxi People’s Hospital Affiliated to Nanjing Medical University, Wuxi, 214023 China; 6 State Key Laboratory of Reproductive Medicine, Nanjing Medical University, Nanjing, 211166 China

**Keywords:** Breast cancer, Metastasis

## Abstract

Dishevelled-associated activator of morphogenesis 1 (DAAM1) is a critical driver in facilitating metastasis in breast cancer (BrCa). However, molecular mechanisms for the regulation of DAAM1 activation are only partially elucidated. In this research, the expression levels of YWHAZ and DAAM1 were examined by immunohistochemistry (IHC) staining in BrCa tissues. The functional roles of tyrosine 3-monooxygenase/tryptophan 5-monooxygenase activation protein zeta (YWHAZ)–DAAM1 axis and their regulator microRNA-613 (miR-613) in BrCa cells and associated molecular mechanisms were demonstrated in vitro. As results, the expression levels of DAAM1 and YWHAZ were significantly upregulated in BrCa tissues compared with normal tissues and remarkably associated with poor prognosis. Besides, DAAM1 and YWHAZ were positively correlated with each other in BrCa tissues. YWHAZ interacted and colocalized with DAAM1 in BrCa cells, which was essential for DAAM1-mediated microfilament remodeling and RhoA activation. Moreover, miR-613 directly targeted both YWHAZ and DAAM1, contributing to inhibiting BrCa cells migration via blocking the complex of YWHAZ–DAAM1. To sum up, these data reveal that YWHAZ regulates DAAM1 activation, and the YWHAZ–DAAM1 complex is directly targeted by the shared post-transcriptional regulator miR-613.

## Introduction

Breast cancer (BrCa) is the most commonly diagnosed malignant neoplasm in females, which has surpassed lung cancer with an estimated 2.3 million new cases in the past year [1]. It has been proved that both environmental and genetic threats contribute to the tumorigenesis of BrCa [2]. However, there is plenty of evidence to support that genetic factors are more directly related to the oncogenesis of cancer. For example, an unconventionally high prevalence of mutations in BRCA1 and BRCA2 (approximately 1–2.5%) among women of Ashkenazi Jewish partially accounts for the high incidence of BrCa in Israel and certain European populations [3]. In clinical practice, BrCa is a heterogeneous disease that contains a wide range of malignancies varying in molecular etiology, cellular biology, imaging appearance, and clinical manifestation [4, 5]. Benefit from earlier screening and enhancement of therapeutic strategies, the prognosis of BrCa patients is significantly improved [6]. However, patients with advanced BrCa accompanied with metastasis still face a risk of death [7]. Thus, control and management of metastasis is the key to improve the prognosis of BrCa patients.

Dishevelled-associated activator of morphogenesis 1 (DAAM1) is a classical actin-associated protein and mediates the polymerization of microfilament, regulating vertebrate gastrulation and cancer metastasis [8, 9]. Previously, we conducted a series of studies on the role of DAAM1 in tumors. DAAM1 is a responder of Wnt5a and collagen IV in the cellular microenvironment, which in turn enhances the activity of RhoA, promoting the invasiveness of BrCa cells [10, 11]. As established regulators, microRNA (miRNA) plays an important role in regulating the expression and function of DAAM1. Studies have shown that miR-208a-5p, miR-34a-5p, and miR-188-5p notably inhibit DAAM1 expression and participate in tumor suppression [12–15]. Besides, the function of DAAM1 is mediated by multiple regulators. Under the action of the secreted Wnt5a, Dishevelled protein competitively binds to the DAD domain of DAAM1 and then the GBD domain is exposed. The naked GBD domain can activate the RhoA and regulate the remodeling of microfilaments [16]. The latest study found that tyrosine phosphorylation at the site of 652 in DAAM1 protein is critical to its molecular function, and the phosphorylation of DAAM1 mediated by the oncogene SRC enhances the invasiveness of lung cancer cells [17].

In the current research, tyrosine 3-monooxygenase/tryptophan 5-monooxygenase activation protein zeta (YWHAZ) was identified as a novel partner of DAAM1, which is essential for the molecular functions of DAAM1. Besides, the interaction of YWHAZ–DAAM1 could be blocked by tumor suppressor miR-613. To sum up, the established miR-613/YWHAZ/DAAM1 axis plays a crucial role in cancer progression, which may be a target to reduce the migration of BrCa cells.

## Results

### DAAM1 and YWHAZ are positively correlated in BrCa

**Fig. 1:**
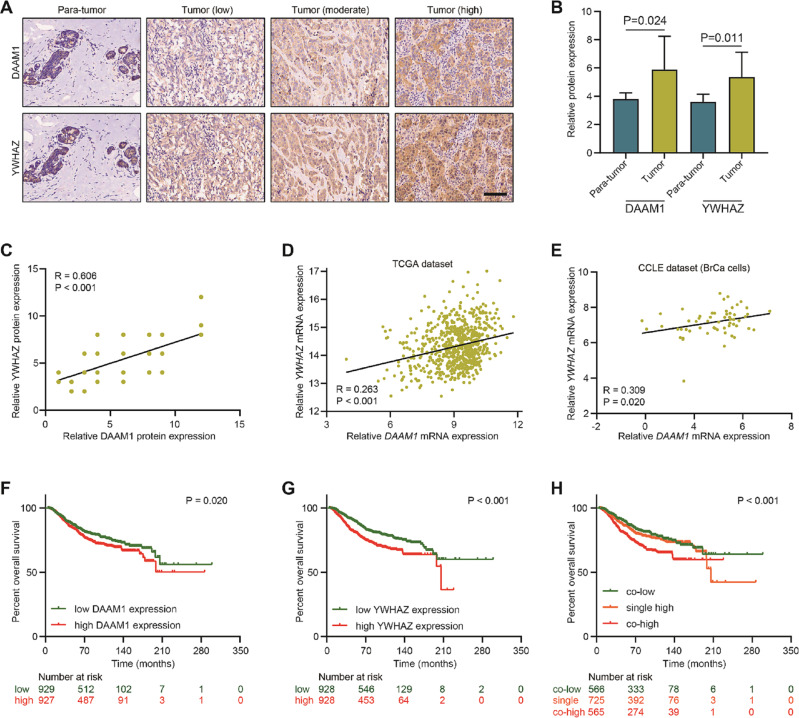
The expression and clinical significance of DAAM1 and YWHAZ in BrCa. **A** Representative microphotographs revealing DAAM1 and YWHAZ expression in para-tumor and tumor with low, medium, and high expression using IHC staining. Magnification: ×200, bar = 100 μm. **B** The expression of DAAM1 and YWHAZ in para-tumor and BrCa tissues. **C** Correlation between the expression levels of DAAM1 and YWHAZ protein in BrCa tissues. **D** Correlation between the expression levels of *DAAM1* and *YWHAZ* mRNA in the TCGA-BrCa dataset. **E** Correlation between the expression levels of *DAAM1* and *YWHAZ* mRNA in a panel of BrCa cells in the CCLE dataset. **F** Kaplan–Meier analysis showed OS curves of patients with high DAAM1 expression vs. low DAAM1 expression in BrCa patients. **G** Kaplan–Meier analysis showed OS curves of patients with high YWHAZ expression vs. low YWHAZ expression in BrCa patients. **H** Kaplan–Meier analysis showed the prognostic value of combined DAAM1 & YWHAZ expression in BrCa patients.

DAAM1 and YWHAZ are both critical drivers in facilitating BrCa metastasis, but the association between DAAM1 and YWHAZ has not been explored. Immunohistochemistry (IHC) staining was performed to examined DAAM1 and YWHAZ expression. As shown in Fig. 1A, the location of DAAM1 and YWHAZ was mainly in the cytoplasm. Besides, compared with para-tumor tissues, the expression levels of DAAM1 and YWHAZ were both upregulated in BrCa tissues (Fig. 1B), which was in accord with previous research [18, 19]. Interestingly, the expression levels of DAAM1 and YWHAZ were positively correlated in the current patients’ cohort (Fig. 1C). To further validate this result, we downloaded the large-scale RNA-seq data from the TGCA and CCLE datasets, and the result showed that DAAM1 was positively correlated with YWHAZ expression in both the TCGA and CCLE datasets (Fig. 1D, E). Moreover, the positive correlation of DAAM1 and YWHAZ was observed not only in BrCa tissues but in most tumor and normal tissues (Tables 1 and 2).

**Table 1:** The correlations between *YWHAZ* and *DAAM1* mRNA expression in the TCGA dataset.

Diseases or Studies	Cases	Coefficient	*P* value
Thymoma	121	0.610	<0.001
Uveal melanoma	79	0.601	<0.001
Head & neck squamous cell carcinoma	564	0.559	<0.001
Kidney chromophobe	91	0.525	<0.001
Prostate adenocarcinoma	548	0.488	<0.001
Uterine corpus endometrioid carcinoma	204	0.488	<0.001
Uterine carcinosarcoma	57	0.472	<0.001
Skin cutaneous melanoma	470	0.459	<0.001
Cervical & endocervical cancer	309	0.436	<0.001
Brain lower grade glioma	523	0.409	<0.001
Diffuse large B cell lymphoma	47	0.408	0.004
Esophageal carcinoma	195	0.401	<0.001
Thyroid carcinoma	571	0.360	<0.001
Sarcoma	264	0.336	<0.001
Kidney papillary cell carcinoma	321	0.327	<0.001
Pancreatic adenocarcinoma	183	0.312	<0.001
Testicular germ cell tumor	154	0.291	<0.001
Cholangiocarcinoma	45	0.268	0.075
Adrenocortical cancer	77	0.256	0.025
Bladder urothelial carcinoma	426	0.256	<0.001
Breast invasive carcinoma	1212	0.248	<0.001
Pheochromocytoma & paraganglioma	185	0.243	0.001
Mesothelioma	87	0.240	0.025
Rectum adenocarcinoma	103	0.217	0.028
Kidney clear cell carcinoma	603	0.165	<0.001
Glioblastoma multiforme	171	0.159	0.038
Ovarian serous cystadenocarcinoma	425	0.125	0.010
Lung adenocarcinoma	574	0.098	0.019
Lung squamous cell carcinoma	548	0.071	0.097
Stomach adenocarcinoma	450	0.043	0.358
Liver hepatocellular carcinoma	421	0.015	0.767
Colon adenocarcinoma	331	−0.045	0.417

**Table 2:** The correlations between *YWHAZ* and *DAAM1* mRNA expression in the GTEx dataset.

Diseases or studies	Cases	Coefficient	*P* value
Kidney tissue	27	0.859	<0.001
Salivary gland	55	0.801	<0.001
Heart mixed tissue	376	0.782	<0.001
Cervix uteri mixed tissue	10	0.709	0.022
Pancreas tissue	165	0.679	<0.001
Bone marrow	70	0.664	<0.001
Blood tissue	444	0.610	<0.001
Liver tissue	110	0.575	<0.001
Prostate tissue	100	0.552	<0.001
Fallopian tube	5	0.540	0.347
Brain mixed tissue	1146	0.534	<0.001
Vagina tissue	84	0.505	<0.001
Stomach tissue	173	0.501	<0.001
Uterus tissue	78	0.459	<0.001
Pituitary tissue	107	0.437	<0.001
Adipose mixed tissue	515	0.390	<0.001
Ovary tissue	88	0.326	0.002
Lung tissue	287	0.305	<0.001
Muscle tissue	396	0.252	<0.001
Breast tissue	179	0.245	0.001
Thyroid tissue	277	0.244	<0.001
Skin mixed tissue	813	0.244	<0.001
Testis tissue	165	0.204	0.009
Nerve tissue	278	0.138	0.021
Spleen tissue	99	0.087	0.394
Adrenal gland	127	0.063	0.485
Esophagus mixed tissue	651	0.051	0.197
Blood vessel mixed tissue	604	−0.041	0.320
Small intestine	92	−0.068	0.520
Bladder tissue	9	−0.373	0.323
Colon tissue	304	−0.479	<0.001

Whereafter, we tried to evaluate the clinical significance of DAAM1 and YWHAZ expression in BrCa. Regrettably, as shown in Table 3, DAAM1 expression was not associated with any clinic-pathological features, but overexpression of YWHAZ was correlated with positive ER, PR, and HER2 status in the current cohort (Table 3). Besides, survival analysis using the large-scale data from the Kaplan–Meier Plotter website indicated that BrCa patients with high expression of DAAM1 or YWHAZ showed shorter overall survival (OS) than those with low expression (Fig. 1F, G). Moreover, co-high DAAM1 and YWHAZ exerted the shortest OS in BrCa (Fig. 1H). Overall, the DAAM1 and YWHAZ are positively correlated with each other and associated with poor prognosis in BrCa.

**Table 3:** The association between DAAM1 and YWHAZ and clinic-pathological features in BrCa.

Features	cases	DAAM1 expression^a^		*χ*^2^ value	*P* value	YWHAZ expression^b^		*χ*^2^ value	*P* value
		Low	High			Low	High		
Age									
≤60	63	26	37	0.164	0.686	29	34	0.173	0.677
>60	22	8	14			9	13		
T stage									
≤2 cm	27	9	18	0.733	0.392	11	16	0.252	0.616
>2 cm	58	25	33			27	31		
N stage									
N0	38	12	26	2.031	0.154	16	22	0.188	0.665
N1–N3	47	22	25			22	25		
ER status									
Negative	21	11	10	1.781	0.182	15	6	8.057	0.005
Positive	64	23	41			23	41		
PR status									
Negative	28	14	14	1.740	0.187	18	10	6.476	0.011
Positive	57	20	37			20	37		
HER2 status									
Negative	27	8	19	1.943	0.163	8	19	3.913	0.048
Positive	57	26	31			30	27		

Note: No patient was diagnosed with distant metastasis in the cohort.

^a^High expression: IRS > 6; low expression: IRS ≤ 4.

^b^High expression: IRS > 6; low expression: IRS ≤ 4.

### YWHAZ interacts with DAAM1

**Fig. 2:**
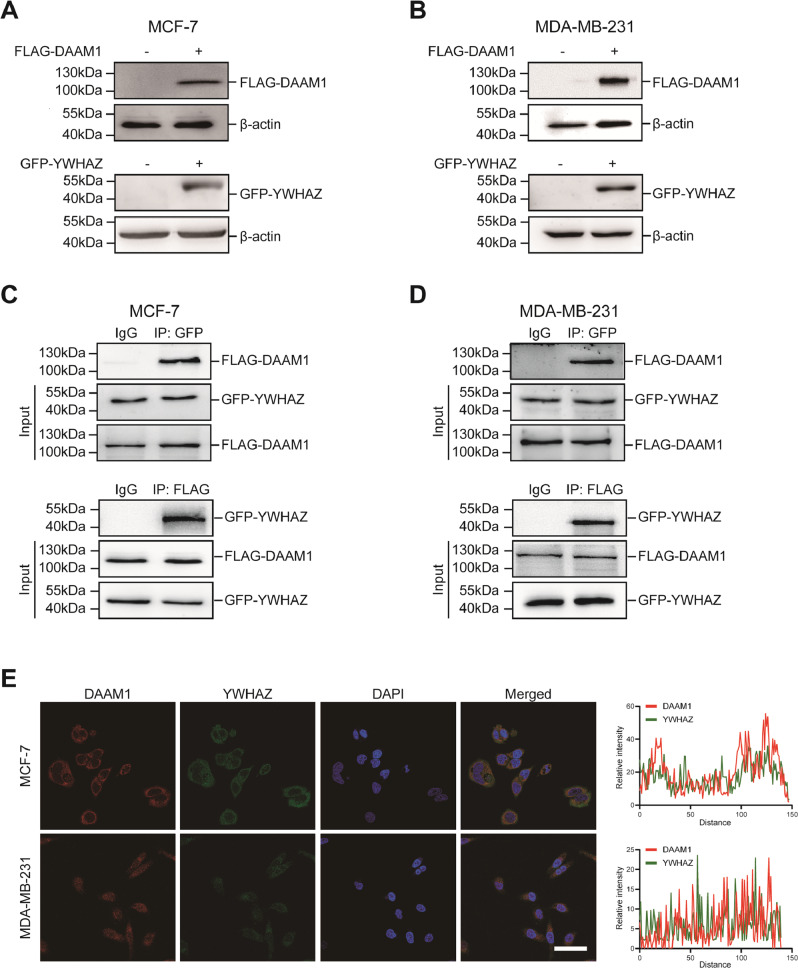
DAAM1 is coprecipitation and colocalized with YHWAZ. **A**, **B** The expression of exogenous proteins in MCF-7 and MDA-MB-231 BrCa cells transfected with the indicated constructs was examined by western blotting. **C**, **D** Endogenous FLAG-DAAM1 and GFP-YWHAZ coimmunoprecipitated with each other with the anti-FLAG or anti-GFP antibody. The whole-cell lysates were analyzed by immunoblotting with the indicated antibodies. **E** Subcellular localization of DAAM1 and YWHAZ was analyzed by immunofluorescence staining followed by capturing using a laser scanning confocal microscope (Olympus FV3000). Magnification: ×600, Bar = 40 μm.

In view of the positive correlation of DAAM1 and YWHAZ, we speculated that YWHAZ could interact with DAAM1. To test this hypothesis, we investigated the physical interaction between DAAM1 and YWHAZ. At first, we constructed full-length DAAM1 and YWHAZ constructs and validated the expression efficiency of the indicated proteins by western blotting in MCF-7 and MDA-MB-231 cells (Fig. 2A, B). MCF-7 and MDA-MB-231 cells expressing endogenous FLAG-DAAM1 and GFP-YWHAZ were lysed, and cell lysates were immunoprecipitated with anti-FLAG or anti-GFP antibodies, followed by immunoblotting. The result suggested that DAAM1 interacted with YWHAZ in BrCa cells (Fig. 2C, D). YWHA proteins have been known to bind to phosphorylated sites of partners [20, 21], and tyrosine phosphorylation at the site of 652 in DAAM1 protein is essential for its function [22]. However, the DAAM1-Y652F and full-length YWHAZ constructs were transfected, the interaction between DAAM1 and YWHAZ was not blocked (Fig. S1). Moreover, immunofluorescence analysis uncovered that DAAM1 and YWHAZ were mainly located in the cytoplasm in both MCF-7 and MDA-MB-231 cells (Fig. 2E). In conclusion, YWHAZ remarkably interacts with DAAM1 in BrCa.

### YWHAZ is essential for BrCa cells migration and the function of DAAM1

**Fig. 3:**
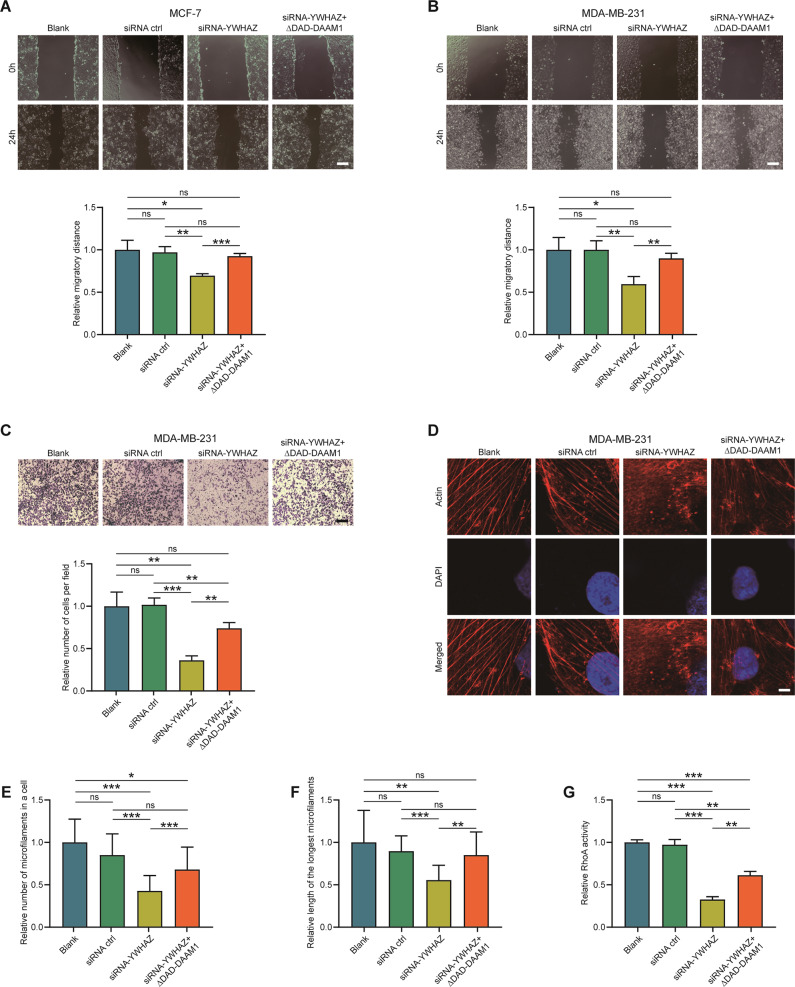
Inhibition of YHWAZ suppresses cell migration and RhoA activity. **A**, **B** The migratory capacity of MDA-MB-231 and MCF-7 cells after transfecting with siRNA-YWHAZ checked by wound-healing assay. Magnification: ×100, bar = 200μm. **C** The migratory capacity of MDA-MB-231 after transfecting with siRNA-YWHAZ checked by Boyden chambers. Magnification: ×100, bar = 200μm. **D** SiRNA-YWHAZ disrupted the formation of microfilaments in MDA-MB-231 cells. The photographs were captured using a laser scanning confocal microscope (Zeiss LSM710). Magnification: ×630. To show the details, additional ×4 was applied. Bar = 2.5 μm. **E**, **F** The number of microfilaments in each MDA-MB-231 cell and the length of the longest microfilament were measured (*n* = 10). **G** RhoA GTPase activation assays showing the downregulated RhoA activity in MDA-MB-231 after transfecting with siRNA-YWHAZ.

Next, siRNA-mediated inhibition of YWHAZ expression was conducted to assess the role of YWHAZ in the migratory capacity of MCF-7 and MDA-MB-231 cells and its effect on the function of DAAM1. First of all, the silencing efficiency of YWHAZ in BrCa cells was confirmed by real-time quantitative reverse transcription PCR (qRT-PCR) and western blotting, respectively (Fig. S2A, B). However, YWHAZ silencing did not change DAAM1 expression and intracellular location (Figs. S3 and Fig. S4). Thus, we speculated YWHAZ silencing only inhibited DAAM1 activation. Compared with the control cells, YWHAZ knockdown MCF-7 and MDA-MB-231 cells showed attenuated migratory capacity revealed by wound-healing assay, and ∆DAD-DAAM1 could reverse the inhibition of migration (Fig. 3A, B). Besides, Boyden chamber assay also was performed using MDA-MB-231 cells and siRNA-transfected cells exhibited decreased migratory capacity, which was reversed by ∆DAD-DAAM1 (Fig. 3C). Considering the binding of YWHAZ and DAAM1, the effect of YWHAZ knockdown on molecular functions of DAAM1 was explored as well. According to previous reports, DAAM1 mediates actin assemblage via modulating RhoA activation through directly recruiting guanine nucleotide exchange factors [23, 24]. YWHAZ knockdown obviously blocked the microfilament formation in MDA-MB-231 cells, and ∆DAD-DAAM1 could repair microfilament damage (Fig. 3D–F). Besides, the activation of RhoA was also inhibited due to YWHAZ knockdown, which was partly reversed by ∆DAD-DAAM1 (Fig. 3G). Collectively, the findings suggest that YWHAZ is essential for the migration of BrCa cells via regulating DAAM1/RhoA signaling.

### MiR-613 negatively regulates YWHAZ and DAAM1

**Fig. 4:**
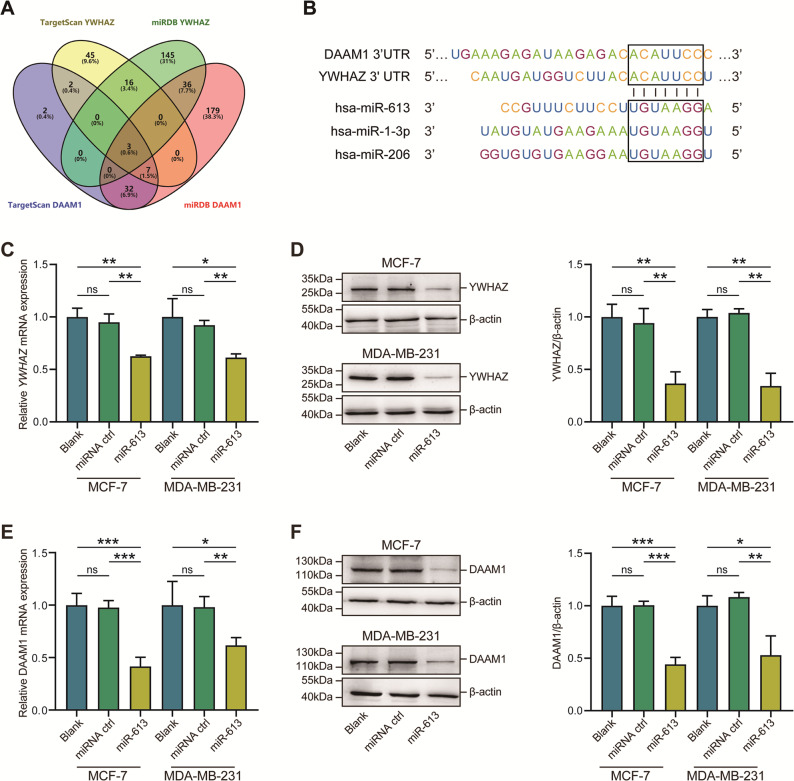
MiR-613 targets YWHAZ and DAAM1 and inhibits their expression. **A** Venn diagram revealing the collective regulatory miRNAs for YWHAZ and DAAM1. **B** Gene structures of YWHAZ and DAAM1 and the binding site of miR-613, miR-1-3p, and miR-206 in the YWHAZ and DAAM1 3′-UTR. **C**, **D** The downregulated YWHAZ both in mRNA and protein levels after miR-613 overexpression in MDA-MB-231 and MCF-7 BrCa cells. β-Actin was used as the loading control. **E**, **F** The downregulated DAAM1 both in mRNA and protein levels after miR-613 overexpression in MDA-MB-231 and MCF-7 BrCa cells. β-Actin was used as the loading control.

Given the positive correlation of DAAM1 and YWHAZ, we speculated that shared regulatory factors may collectively mediate their expression. MiRNA is a type of classical post-transcriptional regulator that downregulates target genes by binding to 3′ untranslated region (3′-UTR). To explore potential miRNAs that targeted both YWHAZ and DAAM1, the TargetScan 7.1 and miRDB tools were used to predict corresponding miRNAs. Consequently, three miRNAs were predicted as collective regulators for YWHAZ and DAAM1, namely miR-613, miR-1-3p, and miR-206 (Fig. 4A). A conserved binding region was predicted to exist in the 3′-UTR of YWHAZ and DAAM1 (Fig. 4B). We next selected miR-613 for further validation. Compared with mammary epithelial cells, MiR-613 was downregulated in BrCa cells, especially in aggressive MDA-MB-231 cells (Fig. S5). Then, miR-613 mimic was transfected into BrCa cells and its effect on YWHAZ and DAAM1 expression was examined. The transfection efficiency of miR-613 mimics overexpression in BrCa cells was confirmed by qPCR (Fig. S6). Notably, miR-613 remarkably inhibited YWHAZ and DAAM1 expression at both mRNA and protein levels (Fig. 4C–F). Taken together, these results show that miR-613 directly targets YWHAZ and DAAM1 and downregulates their expression.

### MiR-613 inhibits BrCa cells migration and RhoA activation

**Fig. 5:**
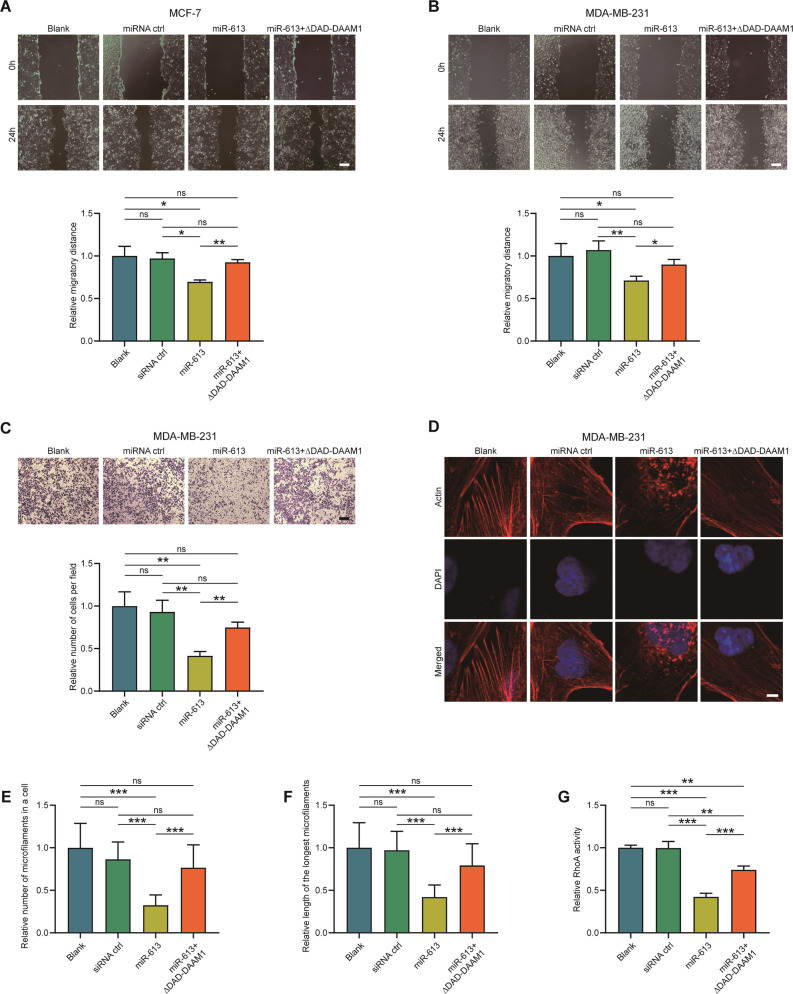
MiR-613 suppresses cell migration and RhoA activity. **A**, **B** The migratory capacity of MDA-MB-231 and MCF-7 cells after transfecting with miR-613 checked by wound-healing assay. Magnification: ×100, bar = 200 μm. **C** The migratory capacity of MDA-MB-231 after transfecting with miR-613 checked by Boyden chambers. Magnification: ×100, bar = 200 μm. **D** miR-613 disrupted the formation of microfilaments in MDA-MB-231 cells. The photographs were captured using a laser scanning confocal microscope (Zeiss LSM710). Magnification: ×630. To show the details, additional ×4 was applied. Bar = 2.5 μm. **E**, **F** The number of microfilaments in each MDA-MB-231 cell and the length of the longest microfilament were measured (*n* = 10). **G** RhoA GTPase activation assays showing the downregulated RhoA activity in MDA-MB-231 after miR-613 overexpression.

We further assessed the role of miR-613 on the migration of BrCa cells. Wound healing and Boyden chamber assays uncovered that miR-613 overexpression suppressed the migratory ability of MCF-7 and MDA-MB-231 cells, and ∆DAD-DAAM1 could reverse miR-613-mediated inhibition of migration (Fig. 5A–C). Besides, whether miR-613 inhibited the formation of microfilaments and the activation of RhoA was investigated. Fluorescent phalloidin was stained to display the arrangement of microfilaments in MDA-MB-231 cells and miR-613 largely disturbed the microfilament assemblage in BrCa cells, and ∆DAD-DAAM1 could repair miR-613-mediated microfilament damage (Fig. 5D–F). Similarly, miR-613 overexpression obviously suppressed RhoA activation, which was partly reversed by ∆DAD-DAAM1 (Fig. 5G). Thus, these results suggest that miR-613 suppresses cell migration via downregulating DAAM1/RhoA axis in BrCa cells.

## Discussion

DAAM1 is a formin-like protein participating in the modulation of actin cytoskeletal rearrangement. Previous studies report that DAAM1 mediates the migration of multiple cancer cells via Rho signaling pathways, including BrCa cells [11], glioblastoma cells [25], melanoma cells [9], ovarian cancer cells [14], esophageal squamous cell carcinoma cells [26], and osteosarcoma cells [27]. Besides, the tyrosine phosphorylation of DAAM1 modulates its homodimer formation and actin polymerization, which is essential for lung cancer invasiveness [17]. Besides, DAAM1 is found to be upregulated in CD133 ^+ ^cancer stem cells, suggesting its critical role in the maintenance of tumor cell stemness [28]. In this research, we validated that DAAM1 was upregulated in BrCa tissues and associated with poor prognosis, which was in accordance with previous observation with a small size of samples [18].

As a novel partner of DAAM1, YWHAZ has been demonstrated to highly express in BrCa tissues and regulate the malignant phenotypes of tumor cells [19, 29]. Overexpression of YWHAZ is proved to be an independent prognostic biomarker for shorter disease-free survival and knockdown of YWHAZ expression by siRNA in BrCa cells significantly inhibits the tumor progression in vitro and in vivo [30]. Besides, YWHAZ is involved in the modulation of stress fibers and focal adhesion in trabecular meshwork cells through regulating the RhoA signaling pathway [31]. Here, we reported that YWHAZ interacted with DAAM1 and highly correlated with DAAM1 expression in BrCa tissues. The known biological functions of DAAM1 mainly depend on mediating the formation of microfilaments through directly regulating the activation of RhoA, which has also been confirmed in BrCa [11, 23, 24]. Importantly, YWHAZ silencing suppressed DAAM1-mediated RhoA activation, explaining the influence of YWHAZ on the microfilament system at least to a certain extent. Tyrosine phosphorylation at the site of 652 in DAAM1 protein has been reported to be essential for its function [17]. However, when the DAAM1-Y652F and full-length YWHAZ constructs were transfected, the interaction between DAAM1 and YWHAZ was not interrupted, suggesting that other tyrosine sites were existing in DAAM1 protein, which might interact with YWHAZ.

Being a hotspot for a long time, many miRNAs have been reported to involve in regulating the oncogenesis and progression of human cancers [32–34]. MiR-613 has been identified as a significant suppressor in multiple cancers, including gastric cancer [35], bladder cancer [36], and BrCa [37]. In BrCa, a penal of targets of miR-613 has been identified, including FAM83A [37], CDK12 [38], and HK2 [39]. YWHAZ has been identified as the target gene of miR-613 in hepatocellular carcinoma [40]. Besides, in our previous research, DAAM1 was also identified as the target gene of miR-613 in triple-negative BrCa [41]. In this report, we uncovered that the complex of YWHAZ and DAAM1 was blocked by miR-613 in BrCa cells, considering that luciferase reporter gene assay has been conducted in HEK-293T cells previously [40, 41]; thus, we did not perform this assay in this research. In addition, miR-613 suppressed the migration and the formation of microfilaments of BrCa cells. Whatever, the current research extended the binding relationship between miR-613 and YWHAZ/DAAM1 to BrCa, particularly ER/PR + BrCa. However, we could not collect fresh BrCa tissue; thus, the expression level of miR-613 was not examined.

**Fig. 6:**
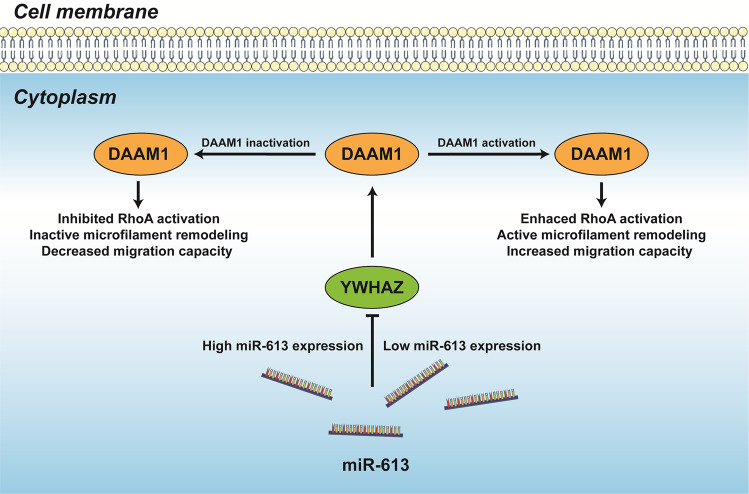
The schematic diagram of the current research. YWHAZ interacts with DAAM1 and is essential for its molecular function. The interaction of YWHAZ and DAAM1 is blocked by miR-613. Thus, the novel axis of miR-613/YWHAZ/DAAM1 could be a target in controlling the cell migration of BrCa cells.

These results manifest that YWHAZ and DAAM1 are upregulated in BrCa tissues compared with normal tissues. Besides, YWHAZ is a novel partner of DAAM1 and essential for the molecular function of DAAM1. In addition, the interaction of YWHAZ and DAAM1 is blocked by miR-613. To sum up, these data suggest miR-613/YWHAZ/DAAM1 axis as a novel target in controlling the migration of BrCa cells (Fig. 6).

## Materials and methods

### Antibodies, plasmids, and reagents

Rabbit polyclonal antibodies against DAAM1 (Cat no. 14879-1-AP), YWHAZ (Cat no. 14881-1-AP), FLAG (Cat no. 20543-1-AP), GFP (Cat no. 50430-2-AP), and β-actin (Cat no. 20536-1-AP) were purchased from ProteinTech (Wuhan, China). Mouse monoclonal antibody against YWHAZ (Cat no. sc-518031) was purchased from Santa Cruz (Dallas, USA). FLAG-full-DAAM1 subcloned into the pcDNA 3.1-FLAG vector has been described previously [42]. FLAG-DAAM1-Y652F vector was generally gifted by Dr. Guang-Chao Chen (National Taiwan University). GFP-tagged YWHAZ were was generated by subcloning YWHAZ into the pEGFP-C2 vector. Constitutive active DAAM1 (∆DAD-DAAM1) was described previously [11]. The purity of all fusion proteins was evaluated by western blotting analysis. The sequence for siRNA-YWHAZ was obtained from a previous report [30]. Small interfering RNA (siRNA) for YWHAZ, negative control, and miR-613 mimics were synthesized by KeyGEN BioTECH (Nanjing, China).

### Tissue microarray

The BrCa tissue microarray section (HBreD090Bc02) was purchased from OutDo BioTECH (Shanghai, China). This section contained 85 BrCa and 5 adjacent normal samples. Relevant records of clinicopathological features were provided by OutDo BioTECH as well. Ethical approval for the usage of the tissue microarray was granted by the Clinical Research Ethics Committee, OutDo BioTECH.

### Immunohistochemistry

IHC staining was performed on the tissue microarray sections. These sections were deparaffinized at 55 ˚C for 30 min, and then washed with xylene for 5 min three times. These sections were rehydrated by successive washes in 100%, 90%, and 70% graded ethanol. Hydrogen peroxidase (0.3%, ZSGB-Bio, Beijing, China) was applied to intercept endogenous peroxidase activity for 20 min. The primary antibodies used in IHC staining were as follows: anti-DAAM1 (1:8000 dilution, Cat no. 14879-1-AP, ProteinTech), anti-YWHAZ (1:3000 dilution, Cat no. 14881-1-AP, ProteinTech), followed by incubation with the corresponding secondary antibodies at room temperature (RT) for 30 min. Immunostained sections were scanned using Aperio Digital Pathology Slide Scanners. The semi-quantitative criterion of evaluating protein levels was described previously [12].

### Cell culture and transfection

MCF-10A and HBL100 mammary epithelial cells, MDA-MB-231 (ER/PR/HER2−) and MCF-7 (ER/PR+) BrCa cells, and HEK-293T cell lines were obtained from the Cell Bank of Chinese Academy of Sciences (Shanghai, China). HBL100, MDA-MB-231, MCF-7, and HEK-293T cells were maintained in high glucose DMEM medium (Hyclone, Thermo Scientific, Waltham, USA) supplemented with 10% fetal bovine serum (Hyclone) at 37 °C with 5% CO_2_. MCF-10A cells were cultured in DMEM/F12 media supplemented with 5% (v/v) horse serum, 20 ng/mL human EGF, 10 μg/mL insulin, 0.5 μg/mL hydrocortisone, penicillin, streptomycin, and 100 ng/mL cholera toxin (Sigma-Aldrich, St. Louis, MO). All cell lines were verified monthly to be mycoplasma negative.

For gene knockdown or overexpression, siRNA, plasmid, miR-613, and negative controls were transfected into MCF-7, MDA-MD-231, or HEK-293T cells using Lipofectamine 2000 according to the manufacturer’s instructions. Transfection efficiency was assessed after 24 h by examining mRNA and protein levels using qRT-PCR and/or western blotting.

### Quantitative real‑time PCR

TRizol reagent (Thermo Fisher Scientific, Waltham, USA) was applied to extract total RNA from BrCa cells. All standardized steps of qRT-PCR were described as previously [12]. The mRNA levels were normalized to the levels of GAPDH. To detect the miR-613 expression level, a total of 1 μg miRNAs were reverse transcripted with Bulge-LoopTM miRNA qRT-PCR Primer Set (Ribobio, Guangzhou, China) and the miR-613 expression was detected with SYBR® Premix Ex Taq™ (TaKaRa, Tokyo, Japan). Primers for miR-613 and U6 snRNA were purchased from Ribobio.

The primers used for gene amplification were as follows: DAAM1: 5′-AAATTGAAACGGAATCGCAAAC-3′ (forward), DAAM1: 5′-GCAAGGCAGTGTAATGAAACG-3′ (reverse); YWHAZ: 5′-TGTAGGAGCCCGTAGGTCATC-3′ (forward), YWHAZ: 5′-GTGAAGCATTGGGGATCAAGA-3′ (reverse); GAPDH: 5′-AGATCATCAGCAATGCCTCCT-3′ (forward), 5′-TGAGTCCTTCCACGATACCAA-3′ (reverse).

### Western blotting analysis

BrCa cells were seeded in 35-mm dishes (6 × 10^5^ cells/dish) and transfected with synthesized siRNA, plasmid, miRNA, or negative control. Forty-eight hours after transfection, total proteins of BrCa cells were harvested using lysis buffer. SDS-polyacrylamide gel electrophoresis and western blotting analysis were next conducted according to the standard protocols. The primary antibodies for FLAG (1:1000 dilution, Cat no. 20543-1-AP, ProteinTech), GFP (1:1000 dilution, Cat no. 50430-2-AP, ProteinTech), YWHAZ (1:1000 dilution, Cat no. 14881-1-AP, ProteinTech), DAAM1 (1:1000 dilution, Cat no. 14879-1-AP, ProteinTech), and β-actin (1:5000 dilution, Cat no. 20536-1-AP, ProteinTech) were applied. Protein levels were standardized to β-actin for each sample.

### Co-immunoprecipitation

The co-immunoprecipitation assay was conducted in standardized steps. Briefly, the cells were washed with ice-cold PBS, lysed in NP-40 buffer containing cocktail, and then centrifuged for 10 min at 12,000 r.p.m. and 4 °C. Primary antibodies or normal rabbit IgG were added to the cell lysate and incubated at 4 °C overnight. The primary antibodies were as follows: anti-FLAG (2 μg, Cat no. 20543-1-AP, ProteinTech) and anti-GFP (2 μg, Cat no. 50430-2-AP, ProteinTech). Then, 15 μL of protein A/G agarose beads was added to each tube, incubated at RT for 3 h, and centrifuged for 3 min at 4000 r.p.m. at 4 °C. A total of 30 μL of 2× SDS-loading buffer was added to the antigen–antibody–protein A/G agarose bead complex, which was boiled for 10 min. The sample was collected for subsequent SDS-polyacrylamide gel electrophoresis and western blotting.

### Bioinformatics analysis

The RNA-sequencing (RNA-seq) data of YWHAZ and DAAM1 in the Cancer Genome Atlas (TCGA) BrCa dataset and survival information were obtained from the Xena website (https://xenabrowser.net/). The gene expression data of YWHAZ and DAAM1 in a panel of BrCa cells were downloaded from the CCLE dataset (https://portals.broadinstitute.org/ccle/). The correlations of YWHAZ and DAAM1 expression in the TCGA and Genotype-Tissue Expression (GTEx) datasets were examined in the ChIPBase v2.0 website (http://rna.sysu.edu.cn/chipbase/) [43]. The prognostic values of YWHAZ and DAAM1 expression in BrCa were examined by the Kaplan–Meier Plotter website (http://kmplot.com/analysis/) [44]. The corresponding miRNAs targeting YWHAZ and DAAM1 were predicted by the TargetScan 7.1 (http://www.targetscan.org/vert_71/) [45] and the miRDB (http://www.mirdb.org/index.html) [46] tools.

### Wound-healing assay

To check migratory capacity, BrCa cells were seeded in 24-well plates (Costar, Corning, NY) and cultured to confluence. The monolayers of cell were wounded by removing the culture insert and washed with PBS to remove cell debris. After 24 h, cells were stained with 0.2% crystal violet for 20 min in RT. The images were photographed at times 0 and 24 h after migration using a Nikon optics microscope conjugated with PowerShot G10 camera (Canon, Tokyo, Japan). The migratory distance was calculated by the minus of the edge of the wound closure between 0 and 24 h.

### Boyden chamber assays

The migratory capacity of MDA-MB-231 cells was further examined in the Boyden chamber (Cat no. 3422, Costar, Corning, NY). The detailed protocol was described as previously [25].

### Immunofluorescence

Coverslips were immersed in a cell medium to allow cells to attach and grow, and then they were washed with PBS three times for 5 min each time. Paraformaldehyde (4%) was applied to fix the cells on the coverslips for 15 min at RT. The coverslips were washed with PBS three times for 3 min each time. Then, the coverslips were incubated with PBS containing 0.5% Triton X-100 for 5 min. Next, the cells were blocked using 5% skim milk for 1 h, after which anti-DAAM1 antibody (1:100 dilution, Cat no. 14879-1-AP, ProteinTech) and anti-YWHAZ antibody (1:100 dilution, Cat no. sc-518031, Santa Cruz) were added. After incubation overnight at 4 °C, the coverslips were washed with PBS once and incubated with corresponding second antibodies (Goat anti-mouse IgG-FITC, Goat anti-rabbit IgG-TRITC, 1:100 dilution, KeyGEN BioTECH) at RT for 1 h. Next, the coverslips were washed with PBS and stained using DAPI. After washing with PBS, the coverslips were sealed with ProLong™ Live Antifade Reagent (Cat no. P36974, Thermo Fisher, USA). Besides, the detailed protocol for actin cytoskeleton staining was described previously [41]. The images were acquired using a laser scanning confocal microscope.

### RhoA GTPase activation assays

Total protein lysates extracted from BrCa cells turned measure RhoA activity by using RhoA GTPase activation assays (Cat no. BK121, Cytoskeleton, Denver, USA). The detailed protocol for detection of RhoA activation was described previously [10].

### Statistical analysis

All statistical analyses were conducted using SPSS 25.0 or GraphPad Prism 8.0. Most of the data were presented as means ± SDs of three independent experiments if not noted and analyzed by Student’s *t*-test. The associations between DAAM1 and YWHAZ expression levels and clinicopathological features were performed using Pearson’s chi-squared test. The correlation between the two indicators was evaluated by Pearson correlation analysis. Log-rank test was conducted to evaluate the difference between the survival curves. Independent prognostic values of DAAM1 and YWHAZ were analyzed by univariate and multivariate Cox analysis. All tests were two-sided performed by SPSS software and *P* ≤ 0.05 was considered statistically significant labeled with * for *P* ≤ 0.05, ** for *P* ≤ 0.01 and *** for *P* ≤ 0.001.

## Supplementary information

Supplementary Figure Legends

Figure S1

Figure S2

Figure S3

Figure S4

Figure S5

Figure S6

## Data Availability

All the data used during the study are available from the corresponding author on request.
