# Supplementary material for: YWHAZ interacts with DAAM1 to promote cell migration in breast cancer
Source: Cell Death Discov. 2021 Aug 27;7:221. doi: 10.1038/s41420-021-00609-7 (PMC8397740; doi:10.1038/s41420-021-00609-7)
Supplement: Supplementary file 1 — Supplementary Figure Legends [file 41420_2021_609_MOESM1_ESM.docx]

**Supplementary Figure Legends**

Figure S1. (A, B) DAAM1-Y652F is coprecipitated with YWHAZ.

Figure S2. The silencing efficiency of YWHAZ in BrCa cells is confirmed by qPCR and western blotting.

(A) SiRNA-YWHAZ significantly downregulated *YWHAZ* mRNA expression in MCF-7 and MDA-MB-231 cells. (B) SiRNA-YWHAZ significantly downregulated YWHAZ protein expression in MCF-7 and MDA-MB-231 cells.

Figure S3. The silencing of YWHAZ did not change DAAM1 expression.

(A) SiRNA-YWHAZ did not change *DAAM1* mRNA expression in MCF-7 and MDA-MB-231 cells. (B) SiRNA-YWHAZ did not change DAAM1 protein expression in MCF-7 and MDA-MB-231 cells.

Figure S4. The silencing of YWHAZ did not change the intracellular location of DAAM1. (A) MCF-7. (B) MDA-MB-231.

Figure S5. Relative expression level of miR-613 in mammary epithelial cells (MCF-10A and HBL100) and BrCa cells (MCF-7 and MDA-MB-231).

Figure S6. The efficiency of miR-613 overexpression in BrCa cells is confirmed by qPCR.
