# Supplementary figures and images for: YWHAZ interacts with DAAM1 to promote cell migration in breast cancer
Source: Cell Death Discov. 2021 Aug 27;7:221. doi: 10.1038/s41420-021-00609-7 (PMC8397740; doi:10.1038/s41420-021-00609-7)

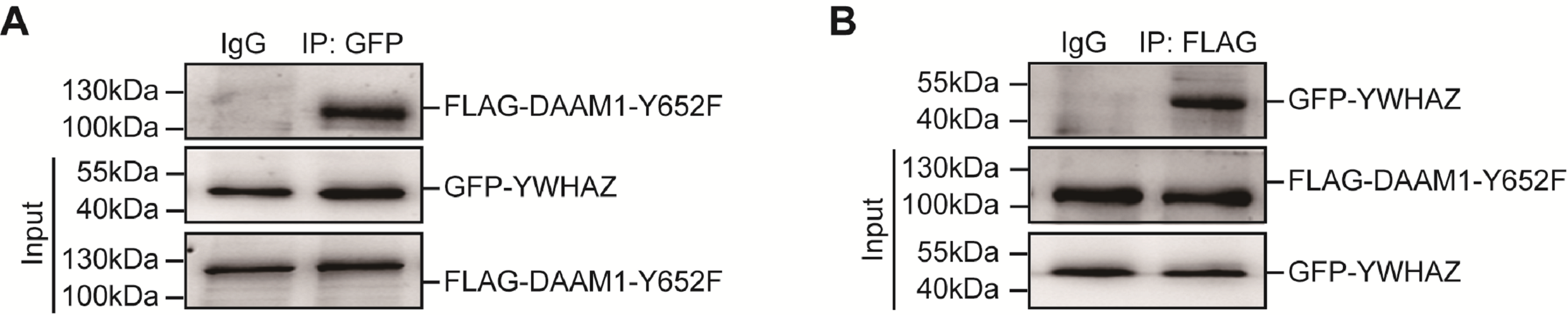

Supplement: Supplementary file 2 — Figure S1 [file 41420_2021_609_MOESM2_ESM.tif]

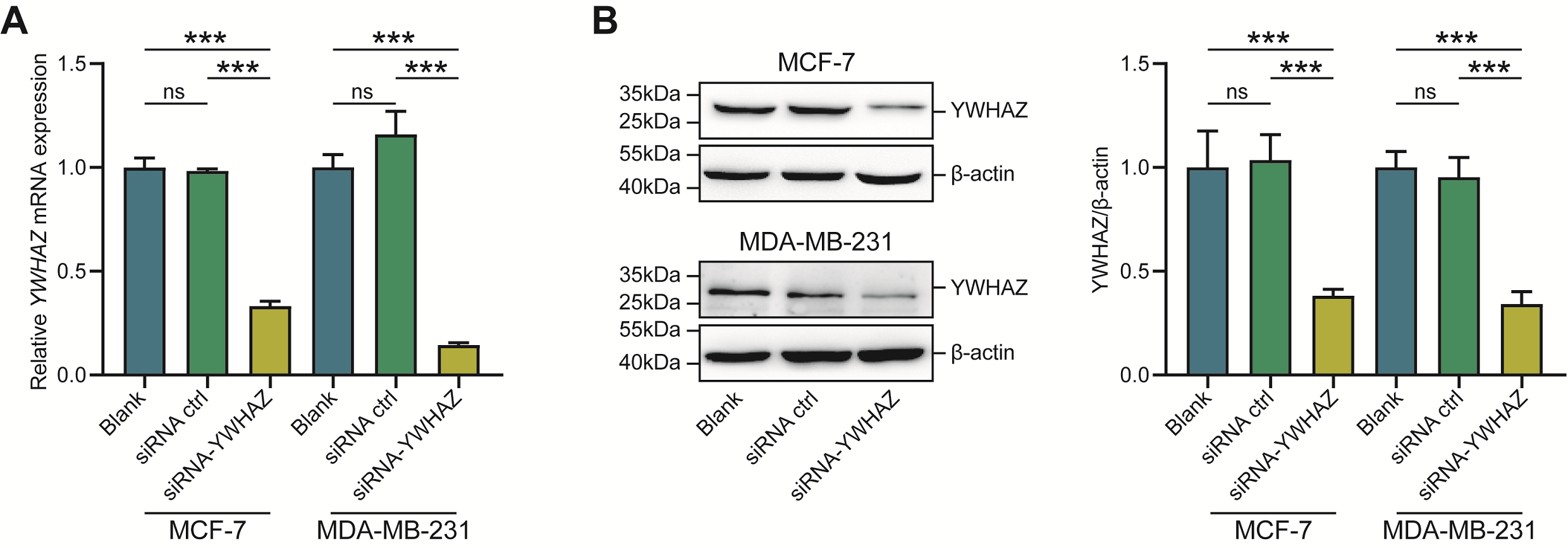

Supplement: Supplementary file 3 — Figure S2 [file 41420_2021_609_MOESM3_ESM.tif]

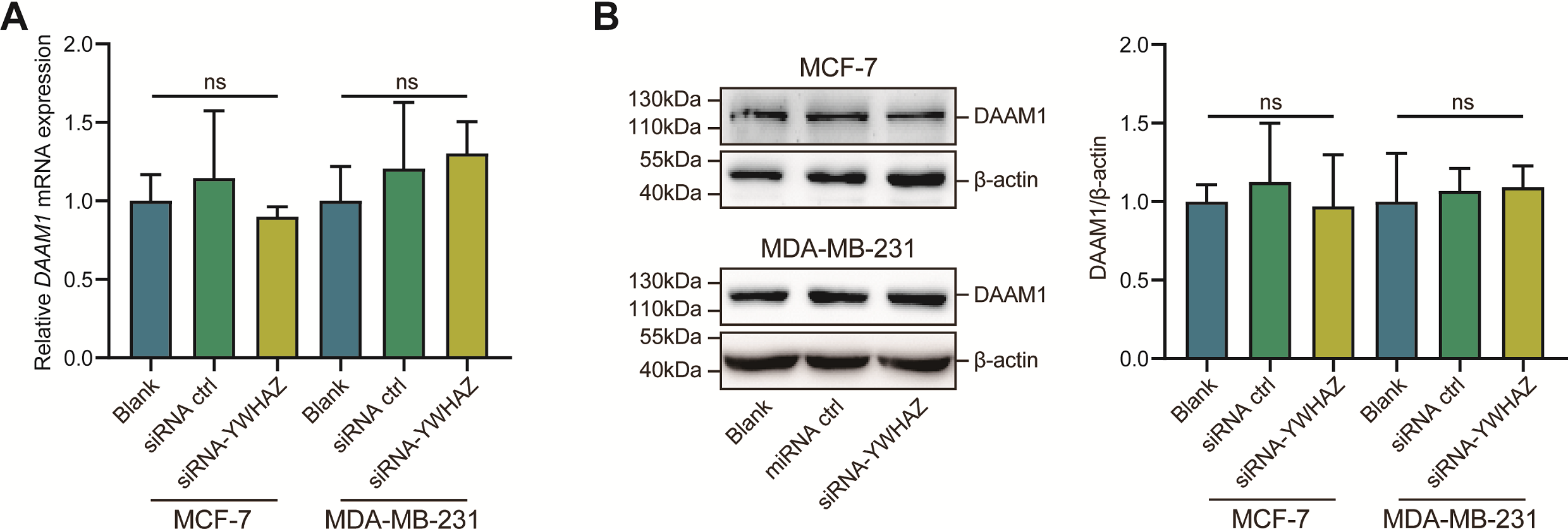

Supplement: Supplementary file 4 — Figure S3 [file 41420_2021_609_MOESM4_ESM.tif]

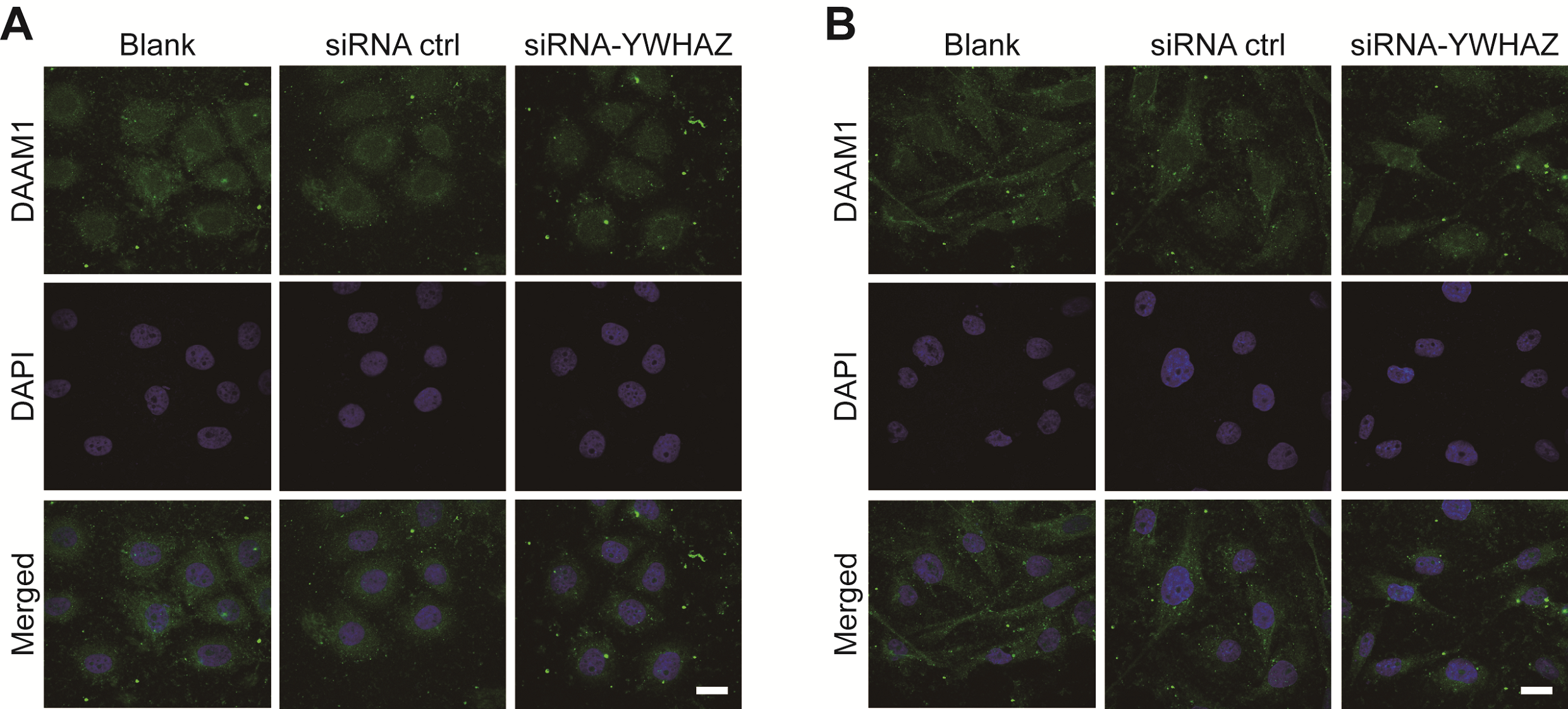

Supplement: Supplementary file 5 — Figure S4 [file 41420_2021_609_MOESM5_ESM.tif]

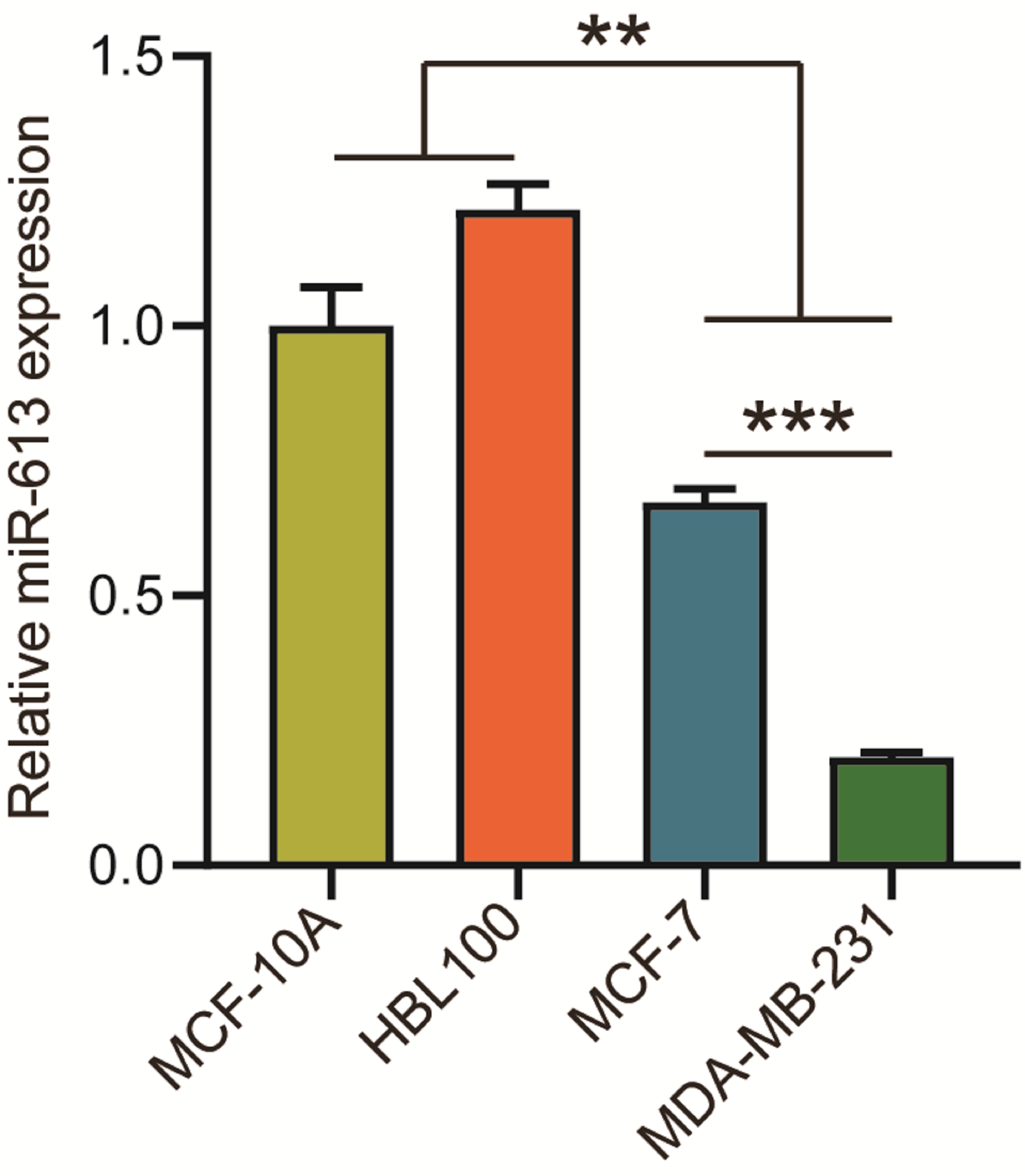

Supplement: Supplementary file 6 — Figure S5 [file 41420_2021_609_MOESM6_ESM.tif]

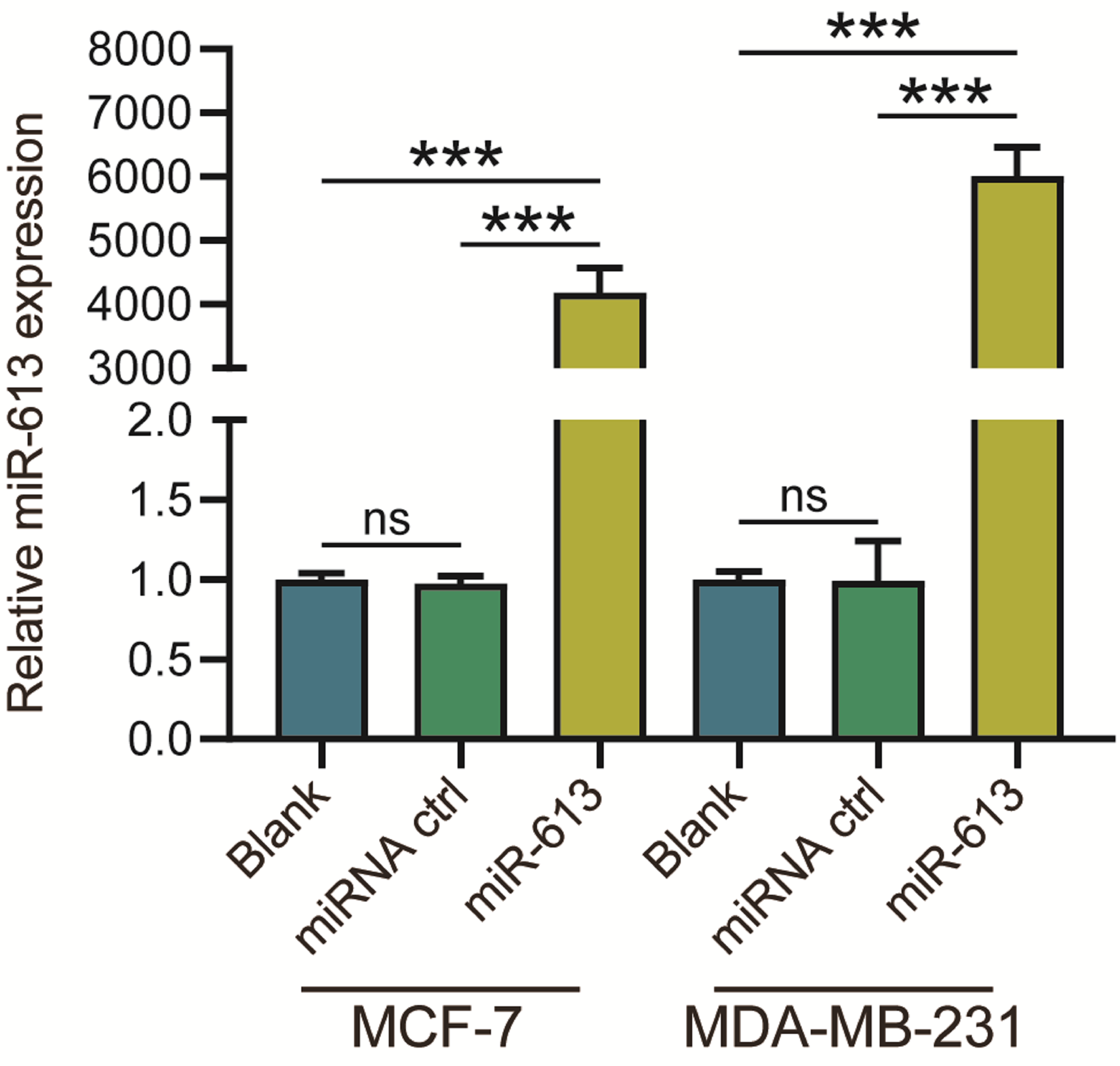

Supplement: Supplementary file 7 — Figure S6 [file 41420_2021_609_MOESM7_ESM.tif]
